# Carcinogenic and non-carcinogenic risk assessment of elemental impurities and bioactive compounds in six wild mushrooms using Monte Carlo simulation

**DOI:** 10.1038/s41598-026-38659-5

**Published:** 2026-03-10

**Authors:** Fadime Canbolat, İsmail Acar, Emine Okumuş, Faruk Ayata

**Affiliations:** 1 Department of Pharmacy Services, Vocational School of Health Services, Çanakkale Onsekiz Mart University, 17100 Çanakkale, Turkey; 2 Department of Organic Agriculture, Başkale Vocational High School, Van Yüzüncü Yıl University, 65080 Van, Turkey; 3 Faculty of Engineering, Department of Food Engineering, Van Yüzüncü Yıl University , 65080 Van, Turkey; 4 Başkale Vocational School, Department of Computer Technologies, Van Yüzüncü Yıl University, 65080 Van, Turkey

**Keywords:** Antioxidant activity, Bioaccumulator, Health risk, Mushroom, Risk element, Cancer, Environmental sciences, Risk factors

## Abstract

Mushrooms, which can bioaccumulate environmental risk elements, have historically been considered a valuable and beneficial food source because of their nutritional and medicinal properties. In this context, the present study aimed to evaluate the natural bioactive potential of mushrooms and the levels of toxic elements. In this study, six edible mushroom species, *Infundibulicybe geotropa*, *Tricholoma populinum*, *Tricholoma scalpturatum*, *Morchella importuna*, *Laccaria laccata*, and *Pholiota carbonaria*, collected from their natural habitats in Bingöl and Van provinces of Türkiye, were investigated. Mushroom samples were collected from 2018 to 2020. The phenolic content and bioactivity of ethanol-extracted mushrooms were analyzed to determine their antioxidant bioactivity profiles. Simultaneously, inductively coupled plasma-mass spectrometry (ICP-MS) was used to evaluate the amounts of cadmium (Cd), lead (Pb), arsenic (As), and mercury (Hg) in the mushroom samples. The accuracy of the ICP–MS measurements was assessed using certified reference materials (CRMs), and recoveries were calculated as the ratio between measured and accredited values, ranging from 87.0% to 92.1%. The results were used to calculate the total carcinogenic risk (TCR), target hazard coefficient (THQ), hazard index (HI), and estimated daily intake (EDI) for adults and children. Monte Carlo simulation was also used to perform a probabilistic risk assessment, taking into account uncertainty in the exposure parameters. Antioxidant activity has been observed in mushroom species. Among mushrooms with antioxidant capacity, *T. scalpturatum* did not appear to pose a non-carcinogenic risk in adults due to elemental impurities (HI < 1); however, this risk increases in children and may pose a health risk (HI > 1). All mushrooms were found to pose a potential carcinogenic health concern (TCR ≥ 1 × 10^− 4^) to adults and children, and their consumption may pose a potential health risk. *L. laccata*, in particular, stands out as the riskiest species for public health, with both low antioxidant activity and high elemental contamination.

**Supplementary Information:**

The online version contains supplementary material available at 10.1038/s41598-026-38659-5.

## Introduction

 Mushrooms are regarded as functional foods due to their high antioxidant capacity and content of phenolic compounds, proteins, fiber, carbohydrates, vitamins, minerals, and other bioactive substances^1–4^. They may offer protection against a variety of chronic illnesses, including diabetes, cancer, heart disease, and neurological conditions. Mushrooms are essential food sources in various aspects of health, culture, and economy in Europe (including Poland, Russia, and Balkan countries), Asia (such as China, Japan, and South Korea), Türkiye, and some Latin American countries. In these regions, there is great interest in mushrooms, as evidenced by the intense consumption of both cultivated and wild edible mushrooms. Despite the presence of bioactive molecules in mushrooms, they can accumulate risk elements from soil as a result of environmental pollution^5–8^. Risk elements occur naturally in the environment or are products of anthropogenic activities^9^. Soil is a significant pathway for contaminating plants and mushrooms. However, the risk of elemental impurity accumulation is particularly high in areas with intensive mining, industrial, and agricultural practices. The bioaccumulation of these potentially harmful components in wild edible mushrooms is further facilitated by anthropogenic activities^10,11^. Mushrooms can rapidly absorb elemental impurities from the soil or atmosphere and are susceptible to environmental factors^12^. The released risk elements accumulate in the environment. They may alter microbial processes, which can increase their availability and toxicity to higher plants, mushrooms, and other organisms, as well as the physicochemical properties of soils, leading to loss of fertility, disturbance of plant metabolism, and reduction in biomass production and crop yields^13–17^. This process can lead to elevated levels of elemental impurities in mushrooms. Thus, even if mushrooms with high levels have high bioactive substance content and antioxidant capacity, chronic toxicity and cancer risk can occur in humans consuming these mushrooms due to elemental impurity exposure^18–20^. Mushrooms, which can bioaccumulate risk elements from the environment, have been historically recognized as valuable and beneficial food sources owing to their nutritional and medicinal properties^8^. Therefore, the levels of risk elements in mushrooms are essential parameters that should be evaluated in terms of food safety and environmental quality.

Saprophytic mushrooms have a high decomposition ability, which increases the concentration of risk elements. Risk elements such as cadmium (Cd), lead (Pb), arsenic (As), and mercury (Hg) can accumulate in the environment and be transmitted to humans through the food chain due to their non-biodegradable properties^21^. These elemental impurities can cause serious issues in the body, including cancer, kidney and liver diseases, and neuropathy^22^. Food safety and health risk assessment have received global attention due to the nature of elemental impurities and their impact on the human food chain^23^. Monitoring the levels of these impurities in food and natural products and assessing the health risks they pose are essential components of food safety. Elements are categorized into three risk categories according to international standards. Following exposure, those that represent a high risk are classified as Class I, and those that represent a moderate or low risk are classified as Classes II and III. The elements Cd, Pb, As, and Hg are all part of Class I^23–28^.

Previous monitoring results and literature publications have indicated that edible mushrooms have greater and more harmful concentrations of Cd, Pb, As, and Hg.^8,29,30^. Therefore, evaluating the risk of contamination in edible mushrooms is crucial for determining food safety. Different types of mushrooms may have different levels and types of elemental impurities^31,32^. As bioaccumulators, mushrooms absorb elemental impurities, particularly those present in the soil. Risk elements such as Cd, Pb, As, and Hg have been detected in mushroom species collected from diverse regions. Numerous studies have documented the potential negative impacts of these elements on human health^29,30,33^. Elemental impurities may vary depending on the type of mushrooms^31,32^. Thus, the variety of mushroom species examined in the literature over the last decade demonstrates the significance of the topic once more^8,13,29,31,33–43^. According to recent research, wild edible mushrooms exhibit significant variations between species, which necessitates species-based evaluations^29,30,33^. Three main criteria were used to choose the species for this study: (i) broad distribution and frequent fruiting in Europe and Anatolia, (ii) consumption in local or public markets or economic value, and (iii) bioactivity and risk of toxic elements (Cd, Pb, As, and Hg). *Infundibulicybe geotropa*, *Tricholoma populinum*, *Tricholoma scalpturatum*, *Morchella importuna*, *Laccaria laccata*, and *Pholiota carbonaria* were the species that were chosen. Studies on the antioxidant bioactivity analysis of these six edible mushroom species and the risk assessment of these elements are not sufficiently represented in the literature.

*I. geotropa* is found in the Bolu and Western Black Sea regions, and phenolic compounds in the methanol extract of its fruiting body have antigenotoxic and antioxidant properties^44^. In Eastern Anatolia, ethnomycological research has included *T. populinum* and *T. scalpturatum* for public consumption; *T. populinum*, in particular, shows great potential for antioxidant action^2^. In contrast, since 2012, *M. importuna* has been grown commercially and rapidly in China’s industrial agriculture, growing from approximately 200 hectares to 16,466 hectares^45^. Although elemental impurities such as Pb, Cd, and As are present in *L. laccata*, data from the Black Sea environment specifically show that the amount of As is significant (e.g., 145 µg g-1 dry weight (dw))^46^. There have been reports of comparatively high amounts of arsenic (As), particularly in *Laccaria* mushroom species, in Europe. *L. laccata* fruit bodies have been reported to contain 147 mg kg^− 1^ dried weight (dw) of As^47^. Additionally, research has demonstrated that the species has strong soil-fungus transfer factors and is an effective Hg accumulator^48^. In post-fire environments, *P. carbonaria* has often been found to be a “pyrophilic” species^49^. The limits for Cd and Pb in mushrooms are set by EU Regulation 2023/915, which emphasizes the need for species-specific risk assessments for public health.

The elemental impurities collected from the environment by mushrooms due to their bioaccumulation properties can also have harmful effects on human health through mushroom consumption. The balance of benefits and harms associated with mushroom consumption is an important consideration. This study makes a unique contribution to the literature by holistically evaluating the antioxidant capacity and elemental impurity analysis in mushrooms using a Monte Carlo-supported probabilistic model. Studies that combine antioxidant profiling and elemental risk analysis in mushrooms are limited or insufficient in Türkiye and the region. In this respect, the study enables a holistic evaluation of fungi in terms of both their natural bioactive potential and their capacity to carry environmental toxic elements. This study aimed to determine the phenolic compounds, antioxidant activity, and contamination levels of elemental impurities, including Cd, Pb, As, and Hg, in six edible mushroom species.

Six edible wild mushroom species—*I. geotropa*, *T. populinum*, *T. scalpturatum*, *M. importuna*, *L. laccata*, and *P. carbonaria*—that were gathered from their natural habitats in the Turkish provinces of Bingöl and Van were the subject of this investigation. The total phenolic, antioxidant, and lipid peroxidation (LPO) inhibitory activities were measured to determine the bioactivity profiles. Inductively coupled plasma mass spectrometry (ICP-MS) was used to assess the amounts of Cd, Pb, As, and Hg in the mushroom samples. The results were used to compute the carcinogenic risk (CR), the total carcinogenic risk (TCR), the target hazard quotient (THQ), the hazard index (HI), and the estimated daily intake (EDI) values for both adults and children. Furthermore, a Monte Carlo simulation was used to perform a probabilistic risk assessment, accounting for exposure. The potential risks associated with mushroom consumption, from both environmental and public health perspectives, are discussed.

## Materials and methods

### Chemicals

Ethanol (C_2_H_5_OH; 99.9%), Folin-Ciocalteu reagent, ethylenediaminetetraacetic acid (EDTA; ≥98%), and sodium carbonate (Na₂CO₃) were sourced from Merck (Darmstadt, Germany). Gallic acid, 2,2-diphenyl-1-picrylhydrazyl (DPPH), ascorbic acid, and 2-acid (TBA) were acquired from Sigma-Aldrich Co. (St. Louis, MO, USA). Nitric acid (HNO_3_, 60%) and hydrogen peroxide (H2O2,≥30%) for assessing elemental impurities were obtained from Sigma-Aldrich (USA). Ultrapure water was used to dilute the sample. Ultrapure water (18 MΩ-cm) was generated using a Milli-Q water purification system (Millipore, Bedford, Massachusetts, USA). For elemental impurity analysis, internal standard solutions (100 µg/mL), tuning solutions (10 µg/mL), and stock solutions (1000 mg/mL) of each element were purchased from Agilent Technologies (USA). NIST 1640a natural water (Gaithersburg, MD, USA) and Strawberry leaves-Trace elements-LGC7162-CRM (LGC, UK) certified reference standards were used for method validation.

### Description of the study area and mushroom sampling

**Fig. 1:**
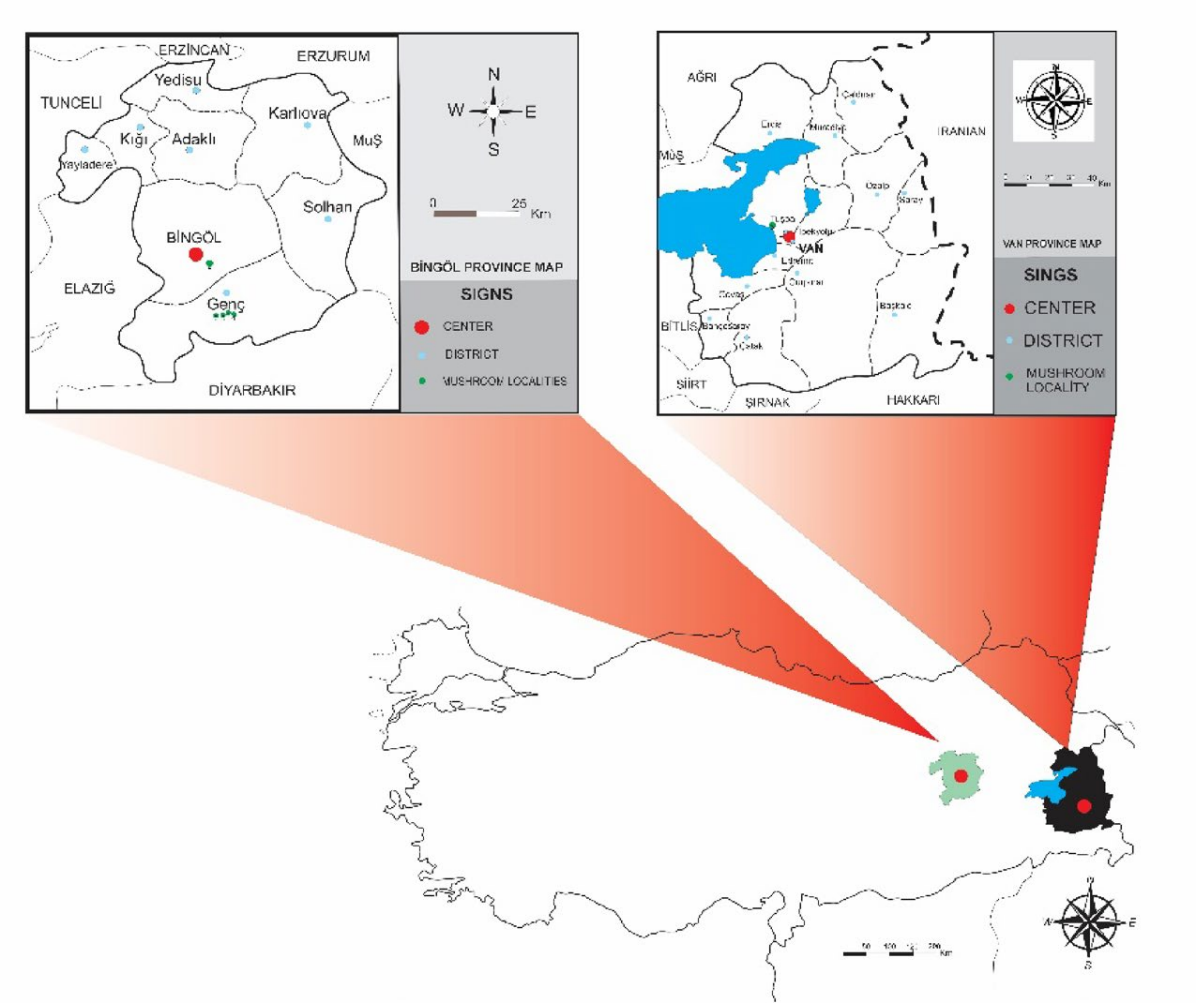
Geographic location of mushroom samples collected in Türkiye. Distribution map of the examined specimens in Türkiye, drawn using CorelDRAW Graphics Suite 2022 (Corel Corporation, Ottawa, Canada).

The specimens were collected between 2018 and 2020 in the Bingöl and Van provinces of Türkiye (Fig. 1). Morphological drawings, distribution maps, and figure plates were prepared and finalized using CorelDRAW Graphics Suite 2022 (Corel Corporation, Ottawa, Canada).

Mushroom specimens were photographed and documented in their natural habitat. In the field, the macromorphological characteristics of the mushrooms were carefully recorded, and adhering soil and dust particles were removed with a fine brush. Specimens of each species were collected from a single population (pooled sampling), and the caps and stems of the individuals were processed together, without separation. The samples were dried at room temperature (approximately 22–25 °C) in a dark, well-ventilated environment until a constant weight was achieved (5–7 days) and subsequently converted into fungarium material. After species identification, specimens that were previously converted into fungarium material were preserved in Van Yüzüncü Yıl University, Faculty of Science, Department of Biology (VANF) fungarium under appropriate conditions. Approximately 100 g of dried fungal material was obtained from pooled individuals for each species. The dried samples were ground in a laboratory mortar to obtain a homogeneous powder. For chemical analyses, three independent biological replicates were prepared per species from the pooled material, and the powders were stored at − 20 °C for up to four weeks until further analysis.

The date and place of collection, coordinates, substrates, and fungarium voucher numbers of the specimens are listed below. Micromorphological features were examined using a Leica DM500 light microscope (Leica, Germany). Species identifications followed Breitenbach & Kränzlin (1984, 1991, 1995), Lamaison & Polese (2005), and Acar et al. (2020)^50–54^.

1. *Infundibulicybe geotropa*

**Specimens examined: **Türkiye, Bingöl, Genç Conifer Forest, 38° 44′35"N, 40° 34′14"E, 1156 m, under *Pinus* sp. trees, 20.05.2018, Acar 1063.

2. *Tricholoma populinum*

**Specimens examined: **Türkiye, Bingöl, Bingöl-Genç Road, Airport neighborhood, 38° 52′21"N, 40° 32′26” E, 1025 m, under *Populus* sp. trees, 24.10.2019, Acar 1124.

3. *Tricholoma scalpturatum*

**Specimens examined: **Türkiye, Van, Yüzüncü Yıl University campus, opposite the Faculty of Business Administration, 38° 34′09"N, 43° 16′59"E, 1661 m, under *Pinus* sp. trees, 15.04.2020, Acar 1190.

4. *Morchella importuna*

Specimens examined: Türkiye, Bingöl, Genç Conifer Forest, 38° 44′38"N, 40° 33′16"E, 1066 m, under *Pinus* sp. trees, 25.03.2018, Acar 1003.

5. *Laccaria laccata*

**Specimens examined: **Türkiye, Bingöl, Genç Conifer Forest, 38° 44′38"N, 40° 33′16"E, 1066 m, under *Pinus* sp. trees, 20.05.2018, Acar 1078.

6. *Pholiota carbonaria*.

**Specimens examined: **Türkiye, Bingöl, Genç Conifer Forest, 38° 44′39"N, 40° 33′10"E, 1057 m, under *Pinus* sp. trees, 20.05.2018, Acar 1071.

### Extraction and in vitro bioactivity analysis

The methods used in our previous study and in the literature were considered in the extraction analysis^30,55,56^. Using a magnetic stirrer, dried powdered mushroom samples (10 g) were extracted in 200 mL of ethanol for 24 h at 45 °C. The supernatant was filtered through Whatman No. 1 coarse filter paper and condensed using a rotary evaporator (Buchi, R-100 model, Flawil, Switzerland) to a total volume of 25 mL. Until additional analysis, the resultant concentrate was stored at + 4 °C.

### In vitro antioxidant activity

The methodologies used in our previous studies and in the literature were considered in the analysis^7,30,57,58^. A vortex was used to thoroughly mix 3.9 mL of DPPH solution (25 mg mL^–1^) with 100 µL of mushroom extract at several concentrations (1–50 mg mL^− 1^). As a negative control, the identical process was performed using ethanol instead of the mushroom extract. A spectrophotometer (Boeco-S22 UV-Vis, Germany) was used to measure the absorbance at 517 nm after the test tubes were left at room temperature in the dark for 60 min.

The data were presented as half-maximal inhibitory concentration (IC_50_; mgmL^− 1^) values, and the percentage inhibition was calculated. Each analysis was performed in triplicate. IC_50_ was determined by expressing the percentage (%) inhibition using the formula given in Eq. (1).

1 $$\% {\text{ }}Inhibition = {\text{ }}\left( {\left( {A_{{control}} - A_{{sample}} } \right)} \right)/A_{{control}} ){\text{ }} \times {\text{ }}100$$

### Total phenolic content (TPC)

The method outlined by Singleton and Rossi (1965) was used to determine the total phenolic content (TPC) of the samples^30,57,59^. First, 150 µL of Folin-Ciocalteu reagent (1:10) was mixed with 50 µL of mushroom extract. The tubes were vortexed after adding 0.8 mL of a 7.5% sodium carbonate (Na_2_CO_3_) solution to the mixture. The absorbance at 765 nm was measured after the samples were incubated for 60 min at room temperature in the dark. The findings were reported as gallic acid equivalent (GAE) per gram dry weight (dw) in milligrams. Every analysis was carried out three times.

### In vitro lipid peroxidation (LPO) inhibition activity

The thiobarbituric acid (TBA) technique was used to evaluate LPO inhibition in the samples. Both our earlier work and the methods used in the literature were considered in the analysis^30,60^. 200 µL of liver tissue homogenate, ascorbic acid, H_2_O_2_ (2.5 mM), ethylenediaminetetraacetic acid (EDTA), and 100 µL of mushroom extract at different doses were mixed. The tubes were incubated at 37 °C in a water bath. Following incubation, the tubes were filled with 30% trichloroacetic acid (TCA) and centrifuged at 4000 × g for 10 min. The supernatant was collected in individual tubes, and 1.5 mL of TBA was added to each tube. The tubes were then heated to 100 °C for 10 min to halt the process. The IC_50_ values were calculated by measuring the absorbance at 532 nm. All analyses were performed in triplicate.

### Elemental impurity analysis in mushrooms

Elemental impurity analyses were performed at the Izmir Katip Çelebi University Central Research Laboratories (IKÇÜ-MERLAB, Türkiye) and Eskisehir Osmangazi University Central Research Laboratory Application and Research Center (ESOGU ARUM, Türkiye). Sample analysis was outsourced to IKÇÜ-MERLAB, which performed elemental impurity analysis using validated methods. NIST 1640a natural water (Gaithersburg, MD, USA) and Strawberry Leaves - Trace Elements - LGC7162-CRM (LGC, UK) standards from Cansever and Söğüt (2025) were used for method validation^61^. The accuracy of the ICP–MS measurements was assessed using certified reference materials (CRMs), and recoveries were calculated as the ratio between measured and accredited values, ranging from 87.0% to 92.1%. The ICP-MS (7800, Agilent Technologies Inc., USA) was used to measure the concentrations of Cd, Pb, As, and Hg in mushroom samples. The instrument conditions and method quantification parameters are listed in Table 1^61^.

Taking into account the elemental impurity analyses in the literature and our previous studies, 8 mL of HNO₃ (60%) and 2 mL of H₂O₂ were added to 0.3 g of dried mushroom samples^23,30,33,62,63^. The samples were allowed to sit in a fume hood for approximately 20 min to facilitate the controlled release of gases before sealing the tubes. The sealed tubes were then digested in a microwave digestion system (Ethos Easy, Milestone Srl., IT) for 30 min. Once microwave digestion was complete, the sample solutions were diluted to a final volume of 30 mL using ultrapure water. ICP-MS was used to analyze the prepared sample solutions. Analyses were performed in triplicate.

**Table 1:** Inductively coupled plasma-mass spectrometry (ICP-MS) instrument conditions and method quantification parameters.

Parameter				Value		
RF Power				1500 W		
RF Voltage				1.80 V		
S/C Temperature				2 °C		
Sample Depth				10 mm		
Nebulizer Gas				1.00 L/min		
Nebulizer Pump				0.10 rps		
Internal standards				^6^Li, ^45^Sc, ^72^Ge, ^89^Y, ^115^In, ^159^Tb, ^209^Bi		
Tune solution				^7^Li, ^89^Y, ^205^Tl		

Quantification parameters						
Element	LOD (µgkg^− 1^)	LOQ (µgkg^− 1^)	Recovery (% from CRM)	Precision (RSD)	Repeatability (RSD)	R^2^
^111^Cd	0.006	0.022	87.0	0.91	1.27	0.999
^208^Pb	0.022	0.074	90.8	0.52	0.71	1.000
^75^As	0.024	0.079	92.1	1.93	3.27	0.999
^201^Hg	0.001	0.003	90.5	5.20	7.20	0.999

LOD limit of detection, LOQ limit of quantification, CRM certified reference material, R^2^ coefficient of determination.

### The carcinogenic and non-carcinogenic human health risk assessment

The human health risk models for carcinogenic and non-carcinogenic effects created by the US EPA have gained recognition and endorsement globally^5,22,23,25,30,33,63^. Risk assessment was conducted for two distinct situations: exposure in adults and that in children. Risk calculations from the literature and our previous studies were taken into account in the calculations^5,12,18,22,23,29,30,33,40,43,63−67^.

### Estimated daily intake (EDI)

The estimated daily intake (EDI) of the pertinent elements (Cd, Pb, As, and Hg) was determined by the daily consumption of the applicable food items, average duration of exposure, and typical concentration levels found in various food samples^30,33^. The EDI was computed based on the dry weight of the mushrooms. Calculations were performed using the formula provided in Eq. (2).

2 $${\text{EDI }}\left( {{\mathrm{mgkg}}^{{ - {\mathrm{1}}}} {\mathrm{day}}^{{ - {\mathrm{1}}}} } \right) = \left( {\left( {{\text{MC }} \times {\text{ IR }} \times {\text{ ED }} \times {\text{ EF}}} \right)/\left( {{\text{ BW }} \times {\text{ AT}}} \right)} \right)$$

EDI refers to the estimated daily intake (mgkg^−1^day^− 1^); MC indicates the concentration of elemental impurities in wild edible mushrooms (mg kg^− 1^); IR denotes the intake rate (6.6 × 10^− 3^ kg person^−1^day^− 1^); EF represents the frequency of exposure (350 days year^− 1^); and ED signifies the duration of exposure (children = 6 years and adults = 26 years). BW is the body weight (children = 25.6 kg; adults = 70 kg); AT for non-carcinogenic risk averages the time as EF × ED (children = 2100 days, and adults = 9100 days), while for carcinogenic risk, it is calculated as 70 years (lifetime) × 365 days year^− 1^ (= 25550 days). Each of the values for these metrics has been adjusted in accordance with the latest US EPA guidelines^22,23,33,63,66,67^.

### Non-carcinogenic risks assessment

#### Target hazard quotient (THQ)

The target hazard quotient (THQ) is used to evaluate the potential health risks associated with human consumption. It determines the extent of non-carcinogenic risks linked to the ingestion of elemental impurities^22,23,33,63,66,67^. The THQ was calculated using Eq. (3).

3 $${\mathrm{THQ}} = {\text{ EDI}}_{{{\mathrm{non}} - {\text{carcinogenic risk}}}} /{\mathrm{RfD}}$$

In this context, EDI refers to the non-carcinogenic risk; EF is the frequency of exposure (350 days year^− 1^); ED indicates the duration of exposure (children = 6 years, adults = 26 years); and BW represents body weight (children = 25.6 kg, adults = 70 kg). To assess non-carcinogenic risk, only the impact during the exposure period was considered, using the AT (averaging time of exposure) EF × ED (children: 2100 days, adults: 9100 days). RfD denotes the oral reference dose for the element measured in mg/kg/day. RfD values for As, Cd, Hg and Pb are 3 × 10^− 4^, 1 × 10^− 3^, 3 × 10^− 4^ and 3.5 × 10^− 3^ mg kg^− 1^ day^− 1^, respectively^12,22,23,30,33,45,63,64,66–68^.

The hazard index (HI) is calculated from the sum of the THQs of all risk elements identified in each mushroom (Eq. 4). The HI is used to evaluate the cumulative risk posed by various elemental impurities.

4 $${\mathrm{HI}} = {\text{ THQ}}_{{{\mathrm{Cd}}}} + {\text{ THQ}}_{{{\mathrm{Pb}}}} + {\text{ THQ}}_{{{\mathrm{As}}}} + {\text{ THQ}}_{{{\mathrm{Hg}}}}$$

An HI value of ≤ 1.0 suggests that there is no significant non-carcinogenic health risk to the population at risk. In contrast, a value of 1.0 < HI < 10.0 signifies a specific non-carcinogenic health risk to the exposed group, and an HI of ≥ 10.0 indicates a significant non-carcinogenic health risk for the population at risk^23,29,30,33,43^.

### Carcinogenic risk (CR) assessment

It is used to evaluate the potential risk of long-term exposure to cancer-causing agents. Because carcinogenic effects are cumulative, they are calculated based on the average daily dose over a lifetime, regardless of whether the individual is a child or an adult. Even a short exposure in childhood can lead to a risk of cancer throughout adulthood, and the effects are spread over a lifetime. Therefore, in the calculation of EDI_carcinogenic risk_, AT (70 years × 365 days = 25,550) is used as a constant value in calculations for children and adults to determine the lifetime risk^66,67^. The CR was calculated using Eq. (5).

5 $${\mathrm{CR}} = {\mathrm{EDI}}_{{{\text{carcinogenic risk}}}} \times {\text{ CSF}}$$

Here, CSF is the oral disposition factor of a carcinogen and is 1.5 (mg kg⁻¹ day⁻¹)⁻¹ for As, 0.0085 (mg kg⁻¹ day⁻¹)⁻¹  for Pb, 6.3 (mg kg⁻¹ day⁻¹)⁻¹ for Cd, and 6.177 (mg kg⁻¹ day⁻¹)⁻¹ for Hg. EF, frequency of exposure (350 days year^− 1^); ED, duration of exposure (children = 6 years, adults = 26 years); BW, body weight (children = 25.6 kg, adults = 70 kg); AT, the averaging period of time a person (70*365 = 25,550 days) used in the calculation^12,33,66,69^.

To assess the total carcinogenic risk (TCR) caused by all elemental impurities detected in a mushroom, the CR values ​​caused by each elemental impurity are summed (Eq. 6).

6 $${\mathrm{TCR}} = {\text{ CR}}_{{{\mathrm{Cd}}}} + {\text{ CR}}_{{{\mathrm{Pb}}}} + {\text{ CR}}_{{{\mathrm{As}}}} + {\text{ CR}}_{{{\mathrm{Hg}}}} ~$$

A CR or TCR value of ≤ 1 × 10^-6^ is considered non-health hazard. CR or TCR values ​​between 1 × 10⁻⁶ and 1 × 10⁻⁴ generally indicate an acceptable or tolerable risk level. However, a CR or TCR value of ≥ 1 × 10⁻^4^ is considered unacceptable and potentially carcinogenic^22,33^.

### Monte Carlo simulation

To examine the possible risk distribution and uncertainty caused by elemental impurity exposure levels, a probabilistic risk assessment was carried out in the literature using the Monte Carlo simulation approach^18,65^. The Monte Carlo simulation method was employed in this study to assess the health hazards associated with elemental impurity exposure, considering the methodologies utilized in the literature. Python 3.9.13 and the following libraries were used to implement the simulation: Matplotlib 3.7.1, Pandas 1.5.3, and NumPy 1.24.4^33^. In the Monte Carlo simulation, probability distributions were selected according to the structures that best represented the actual behavior of the variables. Risk element concentration in different mushroom species with lognormal distribution were considered^70^. The parameters listed in Table 2, which correspond to the values used in the risk analysis, were used to generate the simulation model. The HI for non-carcinogenic health risk and the TCR for carcinogenic health risk were computed after 10,000 iterations of the simulation. Initially, the dataset was loaded. The elemental impurity concentration was randomly sampled from a log-normal distribution, and the body weight was randomly sampled from a normal distribution. With the fixed parameters determined, the EDI was calculated as follows. Using this value, the HI and TCR were calculated as follows. Analyses were performed at the mean and 95th percentile of the results, and visualized with histograms to show the risk levels.

**Table 2:** Health risk assessment parameters related to elemental impurities in mushrooms.

Parameters	Unit	Symbol	Distribution	Value	References
Metal concentration	mg/kg	MC	Lognormal		^43,65,70^
Mushroom intake rate	kg/day	IR	Uniform	0.0033–0.03 kg/day	^22,40,64^
Exposure frequency	days/year	EF	Uniform	350–365	^5^
Exposure duration	year	ED	Uniform	Chid = 6 Adult = 26	^5,22^
Body weight	kg	BW	Normal	Chid=15–49 kg Adult=50–90 kg	^5,22,40,64^
Average time	days	AT	Uniform	EF*ED (for non-carcinogenic) 25,550 (for carcinogenic)	^5,66^

The initial step involved calculating both carcinogenic and non-carcinogenic EDI values based on the parameters presented in Table 2. Subsequently, HI and TCR values were determined utilizing the EDI results. The simulation was executed for 10,000 iterations, allowing for the computation of the HI about non-carcinogenic health risk and TCR related to carcinogenic health risk. Analyses were conducted on both the mean and the 95th percentile of the results, with histograms used to visualize the risk levels.

### Statistical evaluation

To determine the statistical difference in the elemental impurity levels detected in the mushrooms in the study, a One-Way ANOVA test was applied at a 95% confidence interval using the IBM SPSS Statistics 23 program. The experiments were conducted in triplicate. Statistical evaluation was performed using IBM SPSS version 23, starting with a normality test on the dataset. Before the analysis, the Shapiro–Wilk test was applied to each group to determine whether the data showed a normal distribution. The test results yielded *p* > 0.05, indicating that the data were normally distributed and that parametric tests were appropriate for use. Therefore, a one-way analysis of variance was used for intergroup comparisons. One-ANOVA is a commonly used parametric test for comparing mean differences among three or more independent groups. For those elements where notable differences were found between groups in the ANOVA analysis, the Tukey HSD test was employed to identify the specific groups that exhibited these differences. Bioactivity indexes and element load/risk parameter analyses were performed independently of each other because they correspond to different biochemical and environmental processes.

## Result and discussion

### Morphological result

**Fig. 2:**
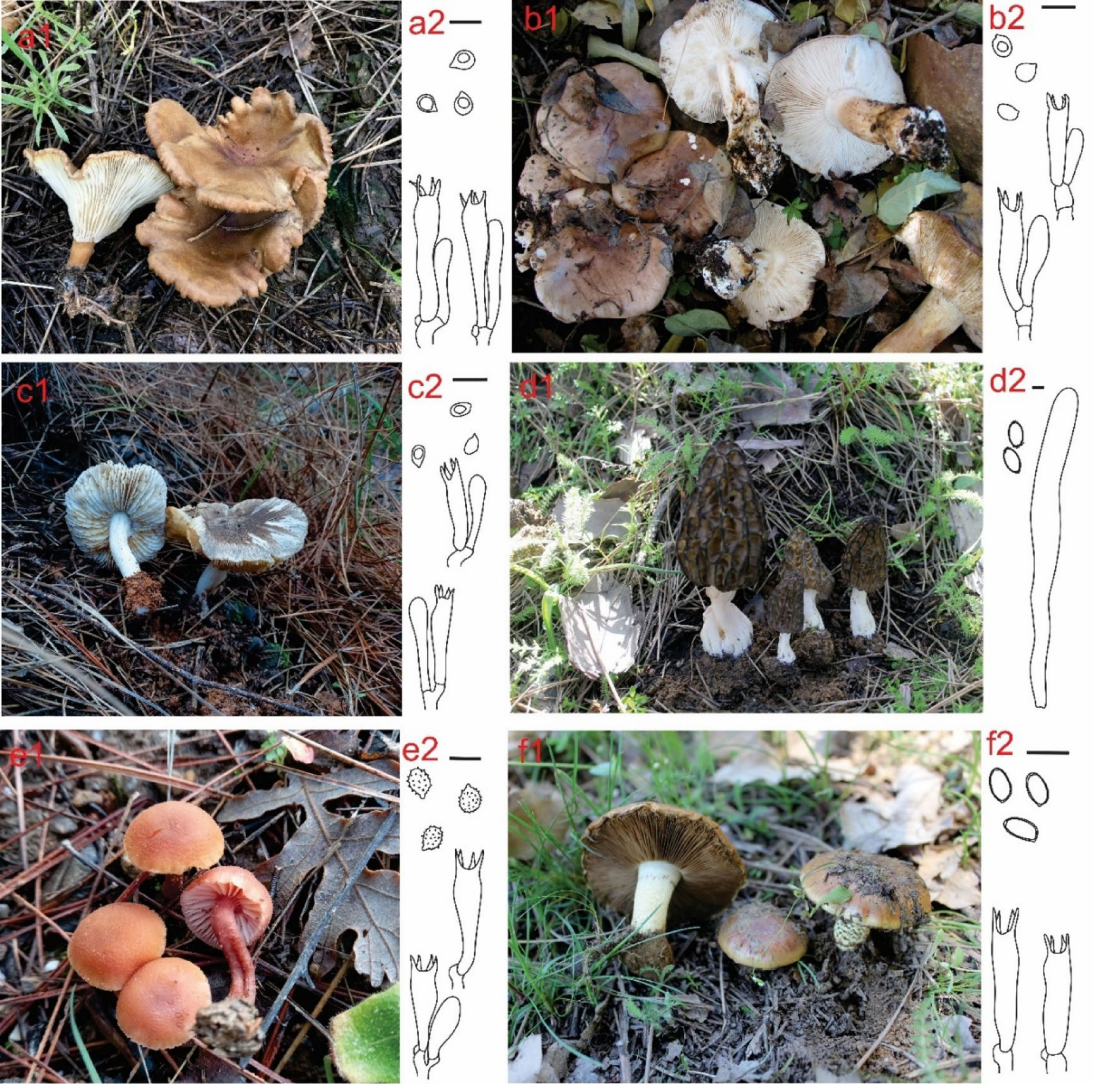
. Macroscopic views of the mushrooms and their microcharacters (a) *Infundibulicybe geotropa*, (b) *Tricholoma populinum*, (c)  *Tricholoma scalpturatum*, (d) *Morchella importuna*, (e) *Laccaria laccata*, (f) *Pholiota carbonaria*  Scale bar: 10 μm.

Macro images of the samples, one of which is a member of Ascomycota and five of which are members of Basidiomycota, and their microcharacters drawn with the CorelDRAW Graphics Suite 2022 (Corel Corporation, Ottawa, Canada) drawing program are presented in Fig. 2.

### Total phenolic content (TPC), antioxidant, and lipid peroxidation (LPO) inhibition activities of the mushrooms

The bioactive compounds in mushrooms provide antioxidant benefits, highlighting their potential for alternative applications, particularly in pharmacology^71^. Table 3 shows the antioxidant activity and TPC values of the mushroom species. The antioxidant activity values of the samples ranged from 8.17 to 19.35 mg mL^− 1^ IC_50_. The highest antioxidant activity was observed in *T. populinum* (*p* < 0.05). The mushroom species *I. geotropa* > *P. carbonaria* > *T. scalpturatum* > *M. importuna* > *L. laccata* (*p* < 0.05). Mushrooms are a significant source of antioxidants, as shown by Islam et al. (2016), who studied 43 different types of mushrooms^72^. Tu et al. (2021)^73^ investigated the DPPH antioxidant activity of *M. importuna* extracted using various drying techniques, and the results ranged from 7.56 to 17.52 mg mL^− 1^. According to Sezgin et al.‘s (2020) investigation into the antioxidant activity of various mushroom species, *T. populinum* exhibited the highest radical scavenging activity of all the species they looked at. This could be due to its high phenolic content^2^. According to the same study, *T. populinum* exhibited greater antioxidant activity than *T. scalpturatum*. According to Heleno et al. (2010)^74^, *L. laccata* has an antioxidant activity of 21.95 mg mL^− 1^. The antioxidant activity of *L. laccata* at a concentration of 250 mg mL^− 1^ was determined to be 71.0% by Volcão et al. (2019)^75^.

**Table 3:** Total phenolic content (TPC), antioxidant, and lipid peroxidation (LPO) inhibition activities of the mushrooms.

Mushroom	DPPH (mgmL^− 1^)	TPC (mg GAEg^− 1^)	LPO (mgmL^− 1^)
*Infundibulicybe* *geotropa*	11.56 ± 0.02^b^	173.57 ± 1.39^e^	13.33 ± 0.27^b^
*Tricholoma* *populinum*	8.17 ± 0.01^a^	209.79 ± 0.	8.28 ± 0.23^a^
*Tricholoma* *scalpturatum*	13.66 ± 0.03^d^	152.29 ± 0.21^c^	15.63 ± 0.06^d^
*Morchella* *importuna*	15.45 ± 0.05^e^	113.53 ± 1.63^b^	17.43 ± 0.13^e^
*Laccaria* *laccata*	19.35 ± 0.04^f^	56.19 ± 1.44^a^	18.17 ± 0.03^f^
*Pholiota* *carbonaria*	12.97 ± 0.02^c^	168.53 ± 0.59^d^	13.82 ± 0.06^c^

Different lowercase letters indicate significant differences (*p* < 0.05). These results were obtained for raw mushrooms (dried and powdered) after extraction.

The phenolic content of mushroom species is closely related to the antioxidant activity of the sample extracts^76^. The TPC values of the examined samples were measured in the range toom of 56.19-209.79 mg GAE g^− 1^ (Table 3). The highest value was found in *T. populinum*, in line with the antioxidant activity results, and the lowest value was found in *L. laccata*. There was a statistically significant difference between the TPC values of the samples (*p* < 0.05). Ao and Deb (2019)^77^ reported that the TPC values of ten wild edible mushroom species ranged from 7.3 to 17.4 mg GAE/100 g. In the literature^74^, the TPC of *L. laccata* was determined to be 1.59 mg GAE g^− 1^. Taşkın et al. (2021)^78^ reported that the TPC values of six different *Morchella* species varied between 135.80 and 281.96 mg GAE g^− 1^. The phenolic content of *shiitake* mushrooms (*Lentinula edodes*) was measured as 176 mg GAE g^− 1^ by Spim et al. (2021)^79^. According to the same study, the high phenolic content of mushrooms makes them suitable for use in various food products, enabling the creation of fortified products. Sezgin et al. (2020) measured the TPC of mushroom samples as 12–195 mg GAE g^− 1^ in polar solvents and 11–69 mg GAE g^− 1^ in apolar solvents^2^. The differences in the bioactive components of the samples may vary depending on many factors, including the geographical region where they were collected, the solvent used, the analysis applied, and the drying method^78^. The formation of intracellular free radicals plays a crucial role in the onset and progression of many diseases. Antioxidants reduce oxidative stress, particularly by inhibiting free radical formation and lipid peroxidation^80^. Therefore, antioxidants and foods rich in antioxidants are crucial for maintaining good health. The LPO inhibition values of the mushroom samples ranged from 8.28 to 18.17 mg mL^− 1^ as IC_50_ (Table 3). The highest activity was observed in *T. populinum* and the lowest in *L. laccata* (*p* < 0.05). There was a similarity between the antioxidant activity of the samples and their anti-LPO activity. Mushrooms exhibit significant potential for use in medicine and pharmacology due to their bioactive components. Although studies on LPO inhibition by mushroom species are pretty limited, the LPO inhibition of *Hebeloma excedens* was determined to be 9.90 mg mL^− 1^ by Okumus (2024)^60^, and the anti-LPO activity of *Infundibulicybe gibba* mushroom was determined to be 6.75 mg mL^− 1^ by Meydan et al. (2025)^81^. Ványolós et al. (2014) reported that *T. populinum* extract showed xanthine oxidase (XO) inhibitory properties, preventing free radical formation and showing a protective effect against cancer^82^. The health benefits of phenolic compounds found in mushroom extracts arise from redox reactions that allow them to function as hydrogen donors in the body. In addition, these components can chelate elements that produce reactive oxygen species. The phenolic content in the structure of mushrooms inhibits various oxidases (monooxygenase, lipoxygenase, etc.) and enzymes, such as glutathione transferase, preventing the formation of free radicals^83^. In recent years, numerous studies have focused on utilizing mushroom extracts to inhibit free radicals and evaluating their therapeutic effects on health^84,85^.

### Elemental impurity risk assessment in mushrooms

Table 4 shows the concentrations of the four elemental impurities (Cd, Pb, As, and Hg) in each mushroom and the correlation levels between them.

**Table 4:** Elemental impurity levels in mushrooms.

Mushroom (Dried)	MC (mgkg^− 1^) mean ± std.dev			
	Cd	Pb	As	Hg
*Morchella importuna*	1.633 ± 1.53^a^	3.058 ± 2.71^a^	2.970 ± 1.87^a^	0.021 ± 0.01
*Tricholoma scalpturatum*	0.928 ± 0.72^b^	1.028 ± 0.87^b^	0.939 ± 0.32^b^	0.033 ± 0.02
*Infundibulicybe geotropa*	3.501 ± 2.15^c^	1.536 ± 0.93^c^	1.840 ± 1.21^c^	0.519 ± 0.32
*Tricholoma populinum*	2.404 ± 1.21^d^	1.467 ± 0.77^d^	2.821 ± 1.14^a^	0.022 ± 0.01
*Pholiota carbonaria*	2.968 ± 1.34^e^	2.187 ± 1.11^e^	1.952 ± 1.32^c^	0.089 ± 0.07
*Laccaria laccata*	10.059 ± 3.24^f^	2.182 ± 1.16^e^	2.000 ± 1.43^c^	ND
Correlations				
Elemental impurity	Cd	Pb	As	Hg
Cd	1	0.188	0.000	– 0.073
Pb	0.188	1	0.652*	– 0.250
As	0.000	0.652*	1	– 0.190
Hg	– 0.073	-0.250	– 0.190	1

MC: elemental impurity concentration. Different lowercase letters indicate significant differences (*p* < 0.05).

*Correlation is significant at the 0.01 level (2-tailed); ND, not detected.

Cd, Pb, As, and Hg can cause severe toxic effects on human health. Cd primarily accumulates in the spleen, liver, and kidneys. It can cause severe damage, especially to the kidneys and liver^86,87^. In addition, it has carcinogenic effects. Typically, the primary source of Cd exposure is food. Specifically, mushrooms can contain high levels of Cd^22^. The Cd levels in the mushrooms included in the study ranged from 0.928 to 10.059 mg kg^− 1^ (Table 4). The highest Cd level was found in *L. laccata*, whereas the lowest Cd level was found in *T. scalpturatum*. In our study, the Cd level in *L. laccata* was 10.059 mg kg^− 1^. Demirbaş et al. (2000)^88^ found the Cd level in *L. laccata* mushrooms collected from different regions to be between 1.07 and 9.65 mg kg^− 1^. The Cd level in our study was close to the upper limit of the Cd levels reported in the literature. Dowlati et al. (2021) reviewed 69 studies on the levels of heavy metals in mushrooms. The review reported that Cd levels ranged from 0.541 to 2.246 mg kg^− 1^. In addition, Chen et al. (2024a)^43^ reported that the Cd level in mushrooms was 26.350 mg kg^− 1^. In their study examining mushrooms as heavy metal biomonitors to detect pollution in forest areas, Świslowski and Rajfur (2018) noted that Cd can accumulate at high levels in mushrooms^89^. Considering both our study data and the literature, the type of mushroom and the places where it grows are effective in Cd levels.

The health impacts of Pb exposure are numerous. Pb exposure can have detrimental effects on the kidneys, blood, and Central Nervous System (CNS)^22^. The Pb levels in the mushrooms included in this study were 1.028–3.058 mg kg^−1^ (Table 4). The highest Pb level was observed in *M. importuna*, whereas the lowest was observed in *T. scalpturatum*. Chen et al. (2024a)^43^ reported that the Pb level in the mushrooms included in their study was 0.022–5.757 mg kg^−1^. The Pb levels in the mushrooms in our study were within the range of those reported for other mushrooms in the literature. When the levels of risk elements in each mushroom and the correlation between them were examined, a significant positive relationship was observed, especially between Pb and As (Table 4). The accumulation of risk elements in mushrooms may vary due to environmental and anthropological factors, such as the soil ecosystem, industrial and agricultural activities (including household waste and heating activities), and the amount of emissions released into the atmosphere, as well as internal factors that may vary depending on the fungal species. Many studies have revealed that mushrooms accumulate Pb, Cd, Hg, and As from the biosphere^31^. The positive correlation observed between Pb and As levels in this study suggests that this may be due to the similarity of the environmental conditions in which the mushroom samples were collected. The positive correlation between Pb and As levels detected in different mushroom species indicates common stress factors within the sample area. Mushroom samples collected in the eastern regions of Türkiye are exposed to regionally similar agricultural (e.g., chemical fertilization, pesticide spraying) and industrial (e.g., traffic emissions, Pb-containing fuel residues, coal, and biomass combustion) activities. Chen et al. (2024b) investigated the levels of heavy metals in polluted soils in China. The study noted that element levels in the soil decreased with distance from polluted areas^87^. Chen et al. (2024a) reported a positive correlation between Pb and As (0.089; *p* < 0.01) in different mushroom species^43^. The study indicated that the correlation between risk elements may be due to similar environmental factors^43^. The literature and our study results suggest that the correlation between risk element types in mushrooms may be influenced by anthropological and ecological factors.

Inorganic arsenic (As) is highly toxic, and its consumption in large quantities can lead to peripheral vascular disease and disorders of the CNS and cardiovascular system. Prolonged exposure to arsenic increases the likelihood of developing lung cancer and is linked to bladder and kidney cancers^22^. In the mushrooms included in this study, the As level was determined as 0.939–2.970 mg kg^−1^ (Table 4). The highest As level was observed in *M. importuna*, whereas the lowest was observed in *T. scalpturatum*. Fu et al. (2020)^22^ reported that As levels in different wild edible mushroom species ranged from 0.16 to 34.5 mg kg^−1^. Although the As levels in the six mushrooms included in our study were similar to those reported in the literature, they were close to the lower limit of the literature levels.

Mercury has a variety of harmful effects on human health. Exposure to Hg can damage the CNS and lungs^22^. Hg levels were detected as 0.021–0.519 mg kg^−1^ in the six mushrooms included in this study (Table 4). The highest Hg level was found in *I. geotropa*, and the lowest was found in *M. importuna*. Hg was not detected in *L. laccata*. Previous studies have reported that the concentration of Hg in edible mushrooms was 0.011–0.768 mg kg^− 1^ in the study of elemental impurity levels in mushrooms^43^. The Hb levels detected in this study were within the range of Hb levels reported in the literature.

Although the harmful health effects of elemental impurities have persisted for a long time in certain regions of the world, exposure to these risk elements continues and is even increasing due to various factors such as industrial activities, pollution, and inadequate waste management^42^. The current study, compared to literature studies on elemental impurities across multiple regions and mushroom species, highlights the importance of differences in elemental impurity levels by area and species (Table 5).

**Table 5:** Comparative findings of elemental impurity levels across several mushroom species and geographical locations

Location	*N*	Cd Range (mgkg^− 1^)	Pb Range (mgkg^− 1^)	As Range (mgkg^− 1^)	Hg Range (mgkg^− 1^)	References
Türkiye	6	0.928–10.059	1.028–3.058	0.939–2.970	0.021–0.519	Current study
Türkiye	5	0.011–4.838	0.495–2.836	3.267–25.823	ND-1.594	^33^
Türkiye	6	0.080–3.370	0.150–1.800	NA	NA	^36^
Türkiye	18	0.280–7.880	ND-5.680	NA	NA	^40^
China	9	0.002–0.961	0.004–0.095	0.029–1.262	ND-0.136	^29^
China	7	0.008–26.350	0.022–5.757	ND-3.037	0.001–2.613	^43^
China	3	0.120–0.180	2.170–3.480	0.370–0.550	NA	^41^
China	11	0.006–48.520	0.007–3.310	0.008–57.340	0.003–0.560	^31^
China	16	0.580–2.880	1.370–10.180	1.090–11.860	NA	^34^
India	3	ND-2.490	0.043-3.500	NA	NA	^35^
Korea	10	0.005-26.0	ND-12.300	ND-18.100	ND-1.730	^38^
Romania	6	3.800–5.100	9.930–12.530	NA	NA	^8^
Slovakia	12	0.670–8.560	3.000-189.000	NA	0.090–4.650	^13^
Poland	7	1.200–84.300	1.300–102.000	NA	NA	^39^
Greece	6	NA	2.800–8.430	NA	NA	^37^

N, number of mushroom species; ND, not detected; NA, not analyzed.

Table 5 shows that elemental impurity levels can reach alarming levels in Asian and European regions. Pollution from elemental impurities has become a pressing concern for both ecology and global public health.

In the current study, although there are mushrooms with high phenolic content and high bioactivity capacity among the six mushroom species (Table 3), when the health risks that may arise from elemental impurity exposure (Table 4) that may occur with the consumption of these mushrooms are examined, it was determined that the main mushroom species that poses a high health risk in adults and children is *L. laccata*. (Table 6).

Table 6 presents the extent of carcinogenic and non-carcinogenic health risks associated with the elemental impurity levels detected in mushrooms for both adults and children. Considering both the phenolic content and the lower antioxidant capacity of *L. laccata* compared to other mushrooms and the health risks that may occur due to elemental impurity exposure, the consumption of this mushroom species will not be beneficial in terms of health when evaluated within the framework of the benefit/harm relationship of preferring this mushroom species due to its bioactivity.

**Table 6:** Results of carcinogenic and non-carcinogenic analysis in mushrooms.

Mushroom (Dried)	Element	EDI_noncarcinogenic risk_ mg kg^− 1^ day^− 1^	THQ	HI	EDI_carcinogenic risk_ mg kg^− 1^ day^− 1^	CR	TCR
Adult							
*Morchella importuna*	Cd	1.539 × 10^− 4^	0.154	1.177	0.548 × 10^− 4^	3.455 × 10^− 4^	5.005 × 10^− 4^
	Pb	2.883 × 10^− 4^	0.082		1.027 × 10^− 4^	8.728 × 10^− 7^	
	As	2.800 × 10^− 4^	0.933		0.997 × 10^− 4^	1.496 × 10^− 4^	
	Hg	0.021 × 10^− 4^	0.007		0.007 × 10^− 4^	4.547 × 10^− 6^	
*Tricholoma scalpturatum*	Cd	0.875 × 10^− 4^	0.087	0.421	0.312 × 10^− 4^	1.963 × 10^− 4^	2.509 × 10^− 4^
	Pb	0.969 × 10^− 4^	0.028		0.345 × 10^− 4^	2.936 × 10^− 7^	
	As	0.885 × 10^− 4^	0.295		0.315 × 10^− 4^	4.731 × 10^− 5^	
	Hg	0.0316 × 10^− 4^	0.011		0.011 × 10^− 4^	6.957 × 10^− 6^	
*Infundibulicybe geotropa*	Cd	3.301 × 10^− 4^	0.330	1.113	1.176 × 10^− 4^	7.408 × 10^− 4^	9.418 × 10^− 4^
	Pb	1.448 × 10^− 4^	0.041		0.516 × 10^− 4^	4.384 × 10^− 7^	
	As	1.736 × 10^− 4^	0.578		0.618 × 10^− 4^	9.273 × 10^− 5^	
	Hg	0.489 × 10^− 4^	0.163		0.174 × 10^− 4^	1.078 × 10^− 4^	
*Tricholoma populinum*	Cd	2.267 × 10^− 4^	0.227	1.160	0.807 × 10^− 4^	5.086 × 10^− 4^	6.558 × 10^− 4^
	Pb	1.383 × 10^− 4^	0.039		0.493 × 10^− 4^	4.188 × 10^− 7^	
	As	2.660 × 10^− 4^	0.887		0.948 × 10^− 4^	1.421 × 10^− 4^	
	Hg	0.0213 × 10^− 4^	0.007		0.007 × 10^− 4^	4.682 × 10^− 6^	
*Pholiota carbonaria*	Cd	2.798 × 10^− 4^	0.279	0.981	0.997 × 10^− 4^	6.279 × 10^− 4^	7.454 × 10^− 4^
	Pb	2.062 × 10^− 4^	0.059		0.734 × 10^− 4^	6.243 × 10^− 7^	
	As	1.841 × 10^− 4^	0.614		0.656 × 10^− 4^	9.836 × 10^− 5^	
	Hg	0.084 × 10^− 4^	0.028		0.029 × 10^− 4^	1.852 × 10^− 5^	
*Laccaria laccata*	Cd	9.484 × 10^− 4^	0.948	1.636	3.378 × 10^− 4^	2.128 × 10^− 3^	2.229 × 10^− 3^
	Pb	2.057 × 10^− 4^	0.059		0.733 × 10^− 4^	6.229 × 10^− 7^	
	As	1.886 × 10^− 4^	0.629		0.672 × 10^− 4^	1.008 × 10^− 4^	
	Hg	NC	NC		NC	NC	
Child							
*Morchella importuna*	Cd	4.210 × 10^− 4^	0.421	3.217	0.346 × 10^− 4^	2.180 × 10^− 4^	3.158 × 10^− 4^
	Pb	7.883 × 10^− 4^	0.225		0.648 × 10^− 4^	5.508 × 10^− 7^	
	As	7.657 × 10^− 4^	2.552		0.629 × 10^− 4^	9.440 × 10^− 5^	
	Hg	0.0565 × 10^− 4^	0.019		0.005 × 10^− 4^	2.869 × 10^− 6^	
*Tricholoma scalpturatum*	Cd	2.392 × 10^− 4^	0.239	1.150	0.197 × 10^− 4^	1.239 × 10^− 4^	1.583 × 10^− 4^
	Pb	2.652 × 10^− 4^	0.076		0.218 × 10^− 4^	1.853 × 10^− 7^	
	As	2.421 × 10^− 4^	0.807		0.199 × 10^− 4^	2.985 × 10^− 5^	
	Hg	0.086 × 10^− 4^	0.029		0.007 × 10^− 4^	4.390 × 10^− 6^	
*Infundibulicybe geotropa*	Cd	9.028 × 10^− 4^	0.902	3.044	0.742 × 10^− 4^	4.675 × 10^− 4^	5.943 × 10^− 4^
	Pb	3.960 × 10^− 4^	0.113		0.325 × 10^− 4^	2.767 × 10^− 7^	
	As	4.746 × 10^− 4^	1.582		0.390 × 10^− 4^	5.852 × 10^− 5^	
	Hg	1.339 × 10^− 4^	0.446		0.110 × 10^− 4^	6.800 × 10^− 5^	
*Tricholoma populinum*	Cd	6.198 × 10^− 4^	0.619	3.172	0.509 × 10^− 4^	3.209 × 10^− 4^	4.138 × 10^− 4^
	Pb	3.783 × 10^− 4^	0.108		0.311 × 10^− 4^	2.643 × 10^− 7^	
	As	7.275 × 10^− 4^	2.424		0.598 × 10^− 4^	8.969 × 10^− 5^	
	Hg	0.058 × 10^− 4^	0.019		0.005 × 10^− 4^	2.954 × 10^− 6^	
*Pholiota carbonaria*	Cd	7.652 × 10^− 4^	0.765	2.681	0.629 × 10^− 4^	3.962 × 10^− 4^	4.703 × 10^− 4^
	Pb	5.638 × 10^− 4^	0.161		0.463 × 10^− 4^	3.939 × 10^− 7^	
	As	5.034 × 10^− 4^	1.678		0.414 × 10^− 4^	6.207 × 10^− 5^	
	Hg	0.230 × 10^− 4^	0.076		0.019 × 10^− 4^	1.168 × 10^− 5^	
*Laccaria laccata*	Cd	25.933 × 10^− 4^	2.593	4.473	132 × 10^− 4^	1.343 × 10^− 3^	1.407 × 10^− 3^
	Pb	5.626 × 10^− 4^	0.160		0.462 × 10^− 4^	3.931 × 10^− 7^	
	As	5.157 × 10^− 4^	1.719		0.424 × 10^− 4^	6.358 × 10^− 5^	
	Hg	NC	NC		NC	NC	

EDI: The estimated daily intake, THQ: The target hazard quotient, HI: The hazard index, CR: Carcinogenic risk, TCR: Total carcinogenic risk, NC: Not calculated.

In adults, the HI value for certain mushroom species is below 1 in non-carcinogenic health risk evaluations, indicating that consumption of these mushrooms is unlikely to present a health hazard (Table 6). The most reliable mushroom species with an HI value less than 1 in terms of health is *T. scalpturatum*. Another mushroom species is *P. carbonaria*, whose HI value is close to the critical threshold but does not exceed it. When the antioxidant capacities of both mushroom species were examined, they ranked in the middle. However, the HI value of *T. populinum*, which had the highest antioxidant activity, was determined to be 1.160 in adults, slightly exceeding the critical threshold (Table 6).

When examining the non-carcinogenic effects in children, the fact that children have lower body weights than adults resulted in higher EDI non-carcinogenic values. High EDI_non−carcinogenic_ values ​​directly affect the increase in HI values, posing a potential health concern to children. As the HI values for all mushroom types exceeded the reference threshold in children, based on body weight, the associated health risks were higher for children than for adults. (Table 6). When the antioxidant capacities of the mushroom species in our study and the potential non-carcinogenic risks associated with exposure to elemental impurities are evaluated together, it appears that *T. scalpturatum* can be safely consumed by adults after short-term exposure (HI; 0.421). However, the non-carcinogenic potential of *T. scalpturatum* in children (HI; 1.150) is higher, and it may pose a health risk to children. When considering the carcinogenic and non-carcinogenic effects of elemental impurities, it is essential to remember that risk elements can pose both non-carcinogenic and carcinogenic risks. Therefore, it is important to investigate not only the non-carcinogenic effects of mushroom consumption but also its carcinogenic effects, ensuring that neither potential risk is significant for healthy consumption of mushrooms.

Since the carcinogenic effects that may occur as a result of elemental impurity exposure through mushroom consumption are cumulative, they can pose a lifelong risk for both children and adults. When the CR or TCR values exceed 1 × 10^− 4^, the risk is considered potentially carcinogenic. The CR or TCR values below 1 × 10^− 6^ are deemed to present no health hazard. The CR or TCR values between 1 × 10^− 4^ and 1 × 10^− 6^ are generally considered to have an acceptable effect on health^22^.

When potential carcinogenic risks due to long-term exposure in adults and children were evaluated, it was observed that Cd exposure was associated with an increased emergence of carcinogenic risks in adults (Table 6). Cd levels in all mushroom species caused CR values to exceed 1 × 10^− 4^ levels. The lowest CR value caused by Cd was in *T. scalpturatum* (CR; 1.963 × 10^− 4^), while the highest CR value was in *L. laccata* (CR; 2.128 × 10^− 3^). However, CR values ​​caused by Pb levels in mushrooms were found to be below 1 × 10^–6^, and Pb is not considered to pose a health hazard. The CR values ​​of As and Hg were found to be between 1 × 10⁻⁶ and 1 × 10⁻⁴, and it is observed that the carcinogenic risk potential is at a tolerable risk level and does not pose a health risk. However, the CR values ​​of As in *M. importuna* (CR; 1.496 × 10^− 4^), *T. populinum* (CR; 1.421 × 10^− 4^) and *L. laccata* (CR; 1.008 × 10^− 4^) and the CR value of Hg in *I. geotropa* (CR; 1.078 × 10^− 4^) (Table 6) being close to the 1 × 10^− 4^ threshold may indicate a shift from tolerable risk to potential health risk. When TCR values were examined for all mushrooms, it was observed that TCR values exceeded 1 × 10^− 4^ and posed a potential health concern in adults. Analysis of the TCR values ​​revealed that *L. laccata* is the riskiest mushroom to consume (Table 6).

Cadmium exposure is a contributing factor to potential health concerns in children. Cd levels in all mushroom species caused the CR values ​​to exceed 1 × 10^− 4^ (Table 6). The lowest CR value caused by Cd was in *T. scalpturatum* (CR; 1.239 × 10^− 4^), while the highest CR value was in *L. laccata* (CR; 1.343 × 10^− 3^). CR values ​​caused by Pb levels in mushrooms were found to be below 1 × 10^− 6^, and Pb is considered not to pose a health hazard. CR values ​​of other elements were within an acceptable range. However, an examination of the TCR values ​​in all mushrooms revealed that TCR values ​​ exceeding 1 × 10^− 4^ pose a potential carcinogenic risk. Analysis of the TCR values ​​revealed that the mushroom with the highest risk of consumption was *L. laccata.*

Although the carcinogenic risk associated with mushroom consumption is evident in both groups, it appears to be more likely in adults. This is due to the higher EDI_carcinogenic_ value, which directly affects carcinogenic risk factors in adults. The lower EDI_carcinogenic_ value in children stems from the differences in ED and BW parameters between adults and children.

Carcinogenic and non-carcinogenic risk assessment is crucial in determining the risks encountered throughout life, depending on the MC, BW, and IR variables in childhood and adulthood, considering the variability of these parameters (Table 2) that may affect the formation of risk and determine carcinogenic and non-carcinogenic health risks. The HI and the TCR levels were evaluated using simulation methods to assess the probabilistic risk dimension of risk element exposure in childhood, adulthood, and the average lifespan (Table 7).

**Table 7:** The probabilistic risk dimension of the elemental impurity exposure in childhood, adulthood, and the average lifespan.

Mushroom	Group	HI 95%	HI 50%	TCR 95%	TCR 50%
*Morchella importuna*	Child	15.395	7.354	1.497 × 10^− 3^	7.221 × 10^− 4^
*Morchella importuna*	Adult	5.711	3.094	2.342 × 10^− 3^	1.313 × 10^− 3^
*Morchella importuna*	Life span	12.808	5.027	1.424 × 10^− 2^	5.771 × 10^− 3^
*Tricholoma scalpturatum*	Child	5.331	2.569	7.468 × 10^− 4^	3.531 × 10^− 4^
*Tricholoma scalpturatum*	Adult	1.948	1.094	1.196 × 10^− 3^	6.445 × 10^− 4^
*Tricholoma scalpturatum*	Life span	4.347	1.757	7.151 × 10^− 3^	2.848 × 10^− 3^
*Infundibulicybe geotropa*	Child	12.149	5.972	2.605 × 10^− 3^	1.200 × 10^− 3^
*Infundibulicybe geotropa*	Adult	4.331	2.506	4.185 × 10^− 3^	2.192 × 10^− 3^
*Infundibulicybe geotropa*	Life span	9.846	4.050	2.488 × 10^− 2^	9.751 × 10^− 3^
*Tricholoma populinum*	Child	15.116	7.172	1.990 × 10^− 3^	9.381 × 10^− 4^
*Tricholoma populinum*	Adult	5.508	3.019	3.177 × 10^− 3^	1.742 × 10^− 3^
*Tricholoma populinum*	Life span	12.410	4.965	1.892 × 10^− 2^	7.543 × 10^− 3^
*Pholiota carbonaria*	Child	12.112	5.946	2.335 × 10^− 3^	1.059 × 10^− 3^
*Pholiota carbonaria*	Adult	4.390	2.511	3.625 × 10^− 3^	1.916 × 10^− 3^
*Pholiota carbonaria*	Life span	9.886	4.083	2.102 × 10^− 2^	8.399 × 10^− 3^
*Laccaria laccata*	Child	20.560	10.211	7.142 × 10^− 3^	3.230 × 10^− 3^
*Laccaria laccata*	Adult	7.492	4.312	1.165 × 10^− 2^	5.933 × 10^− 3^
*Laccaria laccata*	Life span	17.149	7.088	6.930 × 10^− 2^	2.641 × 10^− 2^

HI: The hazard index, TCR: Total carcinogenic risk.

Figure S1-S6 (supplementary material) shows the probabilistic assessment of carcinogenic and non-carcinogenic health risks associated with the consumption of the six mushroom species. When the HI levels of non-carcinogenic effects in six mushroom species were examined in adults (Fig. S1), children (Fig. S2), and throughout the lifespan (Fig. S3), the probabilistic HI value of all mushrooms was found to be greater than one in the mean and 95% level (Table 7). However, the mushroom species with the lowest HI value among the six was *T. scalpturatum*, while the mushroom species with the highest was *L. laccata*. When possible, differences in the population were taken into account in the calculation, and the TCR value due to long-term exposure was found to be approximately 1 × 10^− 4^ in all groups, with most values exceeding 1 × 10^− 4^ (Fig. S4-S6). When risks with a TCR value exceeding 1 × 10^− 4^ were considered a potential health concern, it was probabilistically determined that the long-term effects of consuming these mushroom species in the population posed a potential carcinogenic risk.

These results show that the accumulation of risk elements in mushrooms can cause serious non-carcinogenic health problems in children and adults. Children are more seriously affected by the risk of element exposure than adults, considering their body weight. Protective measures should be implemented for the entire population, particularly children and young people. When examining the Monte Carlo simulation results, evaluating the HI and TCR values for each mushroom species at both the mean and 95% levels provides a better understanding of the probabilistic dimensions of health risks. The mean values reflect the typical risk level predicted for the majority of individuals in the population. In contrast, the 95% level values represent the potential maximum risk that could arise for individuals with the highest exposure probability. In this study, the fact that the HI values for the six mushroom species were generally above one at both the mean and 95% levels indicates the potential for health risks, particularly in sensitive groups (e.g., children). Similarly, the TCR values exceeding 1 × 10 ⁻⁴ at the 95% level indicate that the potential health concern associated with long-term consumption cannot be ignored, even in a small segment of the population. Therefore, it is recommended that public health measures be planned not only based on average risk levels but also by considering the upper limit of the distribution, that is, the 95% level.

The high levels of carcinogenic elements in the mushrooms in our study indicate that long-term consumption may increase the risk of cancer. Additionally, the presence of non-carcinogenic substances that may have harmful effects through bioaccumulation should be taken into account. While these results demonstrate that the mushrooms used in this study possess varying degrees of antioxidant properties, exposure to risk elements resulting from consuming these mushrooms could pose a significant threat to public health, and their consumption should be carefully evaluated in future studies. In light of this information, it is recommended that the potential health risks of mushrooms, which are commonly consumed and collected in Türkiye and globally, be explained to local people, and that they be consumed responsibly. It is also recommended that relevant institutions intensify quality control over mushroom species sold in the market.

### Limitations

The ethanol used in the study for mushroom extraction was preferred because it is a safe, non-toxic solvent that dissolves phenolic compounds effectively, does not interfere with analyses, is environmentally friendly, and is suitable for use in the food/pharmaceutical fields. Further research is needed on the recovery of phenolic compounds using methanol or different solvent combinations.

## Conclusion

This study demonstrated that mushrooms are significant sources of biologically active chemicals and markers that require close monitoring for the accumulation of environmental contaminants, particularly elemental impurities. Among the six mushroom species examined, *T. scalpturatum*, which possesses antioxidant capacity, appears not to pose a non-carcinogenic risk in adults; however, this risk may be increased and could occur in children. However, because all mushrooms have carcinogenic potential in both children and adults, their consumption has been identified as a potential health risk. *L. laccata*, in particular, stands out as the riskiest species for public health, with both low antioxidant activity and high elemental contamination. Monte Carlo simulations reinforced these findings. The obtained data show that mushrooms collected from the natural environment and directly consumed can effectively transfer environmental pollutants to the food chain; in this context, it is a severe warning for consumer health. In this study, bioactivity data of wild mushrooms in Türkiye were integrated with elemental pollution risk data, contributing to health risk assessment studies as regional data.

By establishing a correlation between elemental impurity loads and the bioactive potential of edible wild mushroom species, our study provides critical new insights into how mushrooms interact with forest ecosystems and human health. We emphasize that the ecological functions of mushrooms should be considered in conjunction with their potential toxicological hazards by integrating antioxidant profiling with probabilistic health risk assessment. In addition to highlighting the necessity of carefully assessing mushroom resources in light of biodiversity protection, climatic resilience, and safe economic usage, this viewpoint supports the sustainable integration of wild mushrooms into forestry and food systems. In this respect, the study has interdisciplinary value, as it prepares the scientific ground not only for the fields of mycotoxicology and environmental health, but also for food safety, toxicology, and public health policies. Supporting such studies with a broader range of species and geographical regions is crucial for developing safe consumption practices and environmental risk maps.

## Supplementary Information

Below is the link to the electronic supplementary material.

Supplementary Material 1

## Data Availability

All data generated or analysed during this study are included in this published article.
