# Supplementary material for: Carcinogenic and non-carcinogenic risk assessment of elemental impurities and bioactive compounds in six wild mushrooms using Monte Carlo simulation
Source: Sci Rep. 2026 Mar 10;16:11755. doi: 10.1038/s41598-026-38659-5 (PMC13061964; doi:10.1038/s41598-026-38659-5)
Supplement: Supplementary file 1 — Supplementary Material 1 [file 41598_2026_38659_MOESM1_ESM.docx]

**Carcinogenic and non-carcinogenic risk assessment of elemental impurities and bioactive compounds in six wild mushrooms using Monte Carlo simulation**

Fadime Canbolat^1^,*, İsmail Acar^2^, Emine Okumuş^3^, Faruk Ayata^4^

**1**Department of Pharmacy Services, Vocational School of Health Services, Çanakkale Onsekiz Mart University, 17020, Çanakkale, Türkiye, <https://orcid.org/0000-0001-6759-7735>

**2**Department of Organic Agriculture, Başkale Vocational High School, Van Yüzüncü Yıl University, 65080, Van, Türkiye, https://orcid.org/0000-0002-6049-4896

3Faculty of Engineering, Department of Food Engineering, Van Yüzüncü Yıl University, 65080, 65080, Van, Türkiye, https://orcid.org/0000-0001-5266-8633

4Başkale Vocational School, Department of Computer Technologies, Van Yüzüncü Yıl University, Van, 65080, Van, Türkiye, https://orcid.org/0000-0003-2403-3192

***Correspondence:** Fadime Canbolat; [fadime.canbolat@comu.edu.tr](mailto:fadime.canbolat@comu.edu.tr)

**Supplementary Material**

**
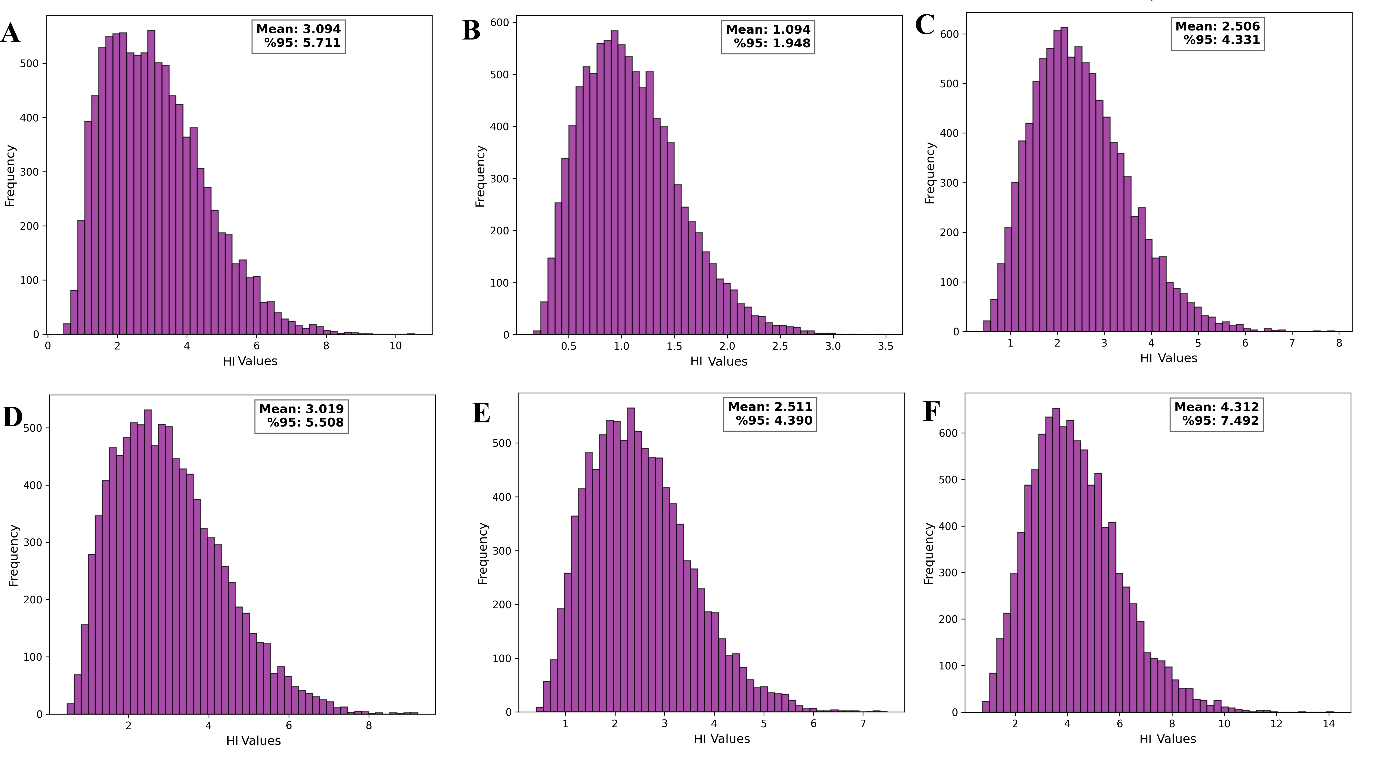
**

**Fig. S1.** Non-carcinogenic risk assessment in adults by monte carlo simulation. A) HI*-Morchella importuna,* B) HI*-Tricholoma scalpturatum,* C) HI*-Infundibulicybe geotropa*, D) HI*-Tricholoma populinum*, E) HI*-Pholiota carbonaria,* F) HI*-Laccaria laccata*

**
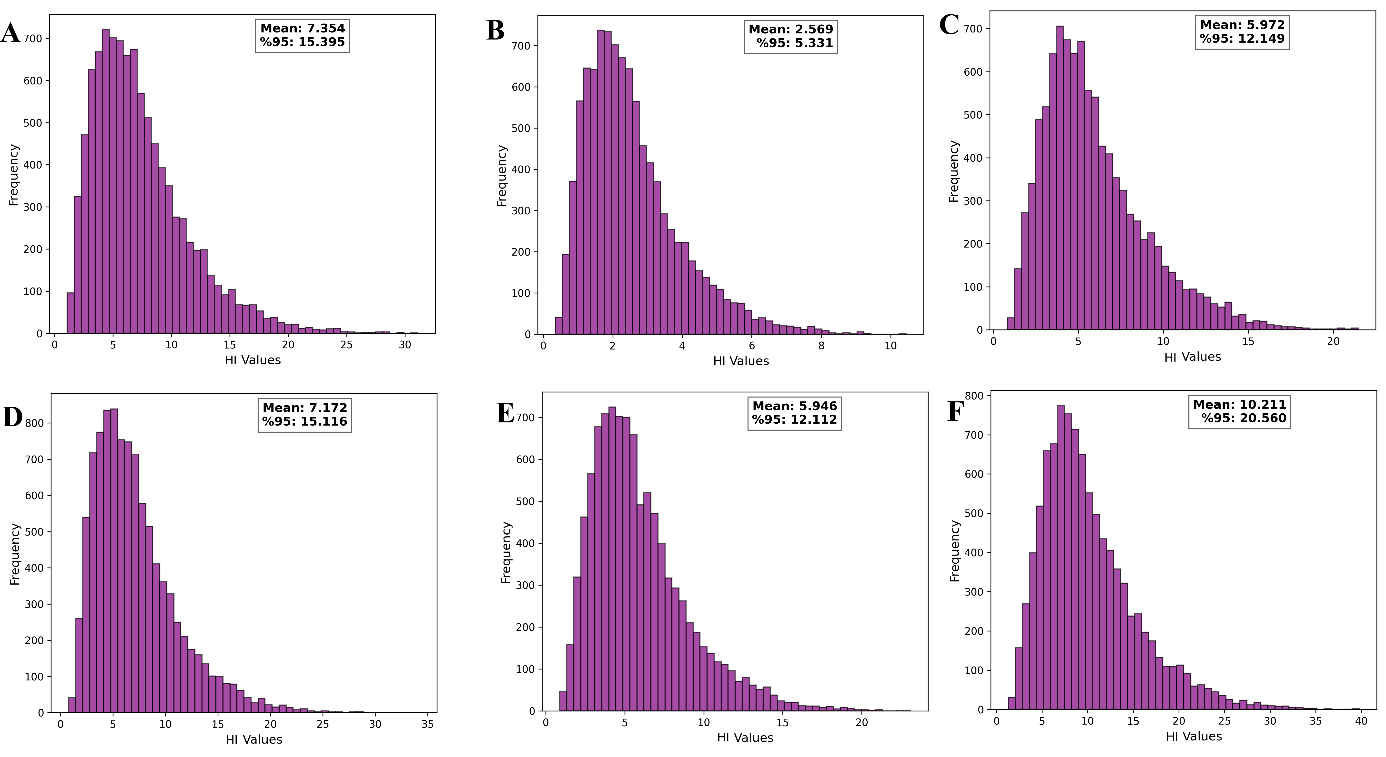
**

**Fig. S2.** Non-carcinogenic risk assessment in children by monte carlo simulation. A) HI*-Morchella importuna,* B) HI*-Tricholoma scalpturatum,* C) HI*-Infundibulicybe geotropa*, D) HI*-Tricholoma populinum*, E) HI*-Pholiota carbonaria,* F) HI*-Laccaria laccata*


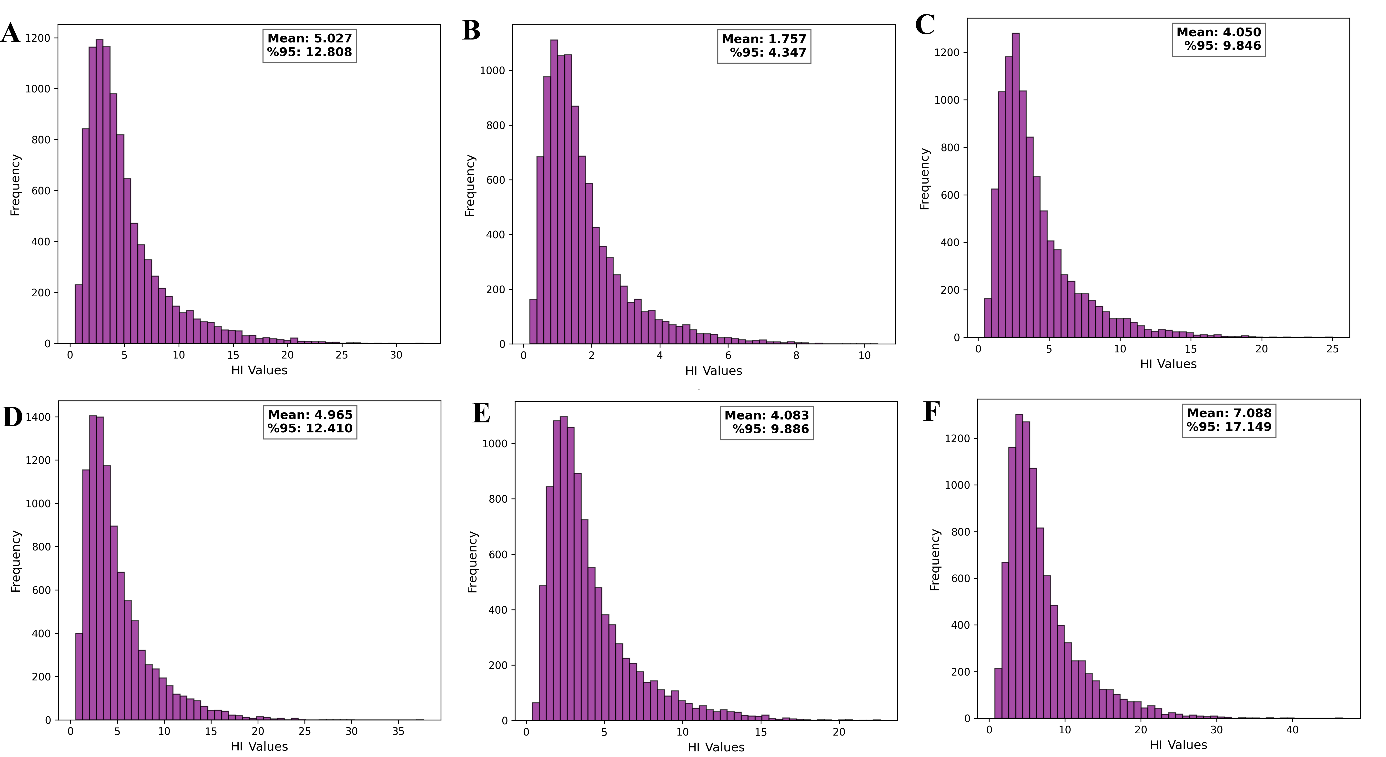


**Fig. S3.** Non-carcinogenic risk assessment throughout the life span by monte carlo simulation. A) HI*-Morchella importuna,* B) HI*-Tricholoma scalpturatum,* C) HI*-Infundibulicybe geotropa*, D) HI*-Tricholoma populinum*, E) HI*-Pholiota carbonaria,* F) HI*-Laccaria laccata*

*
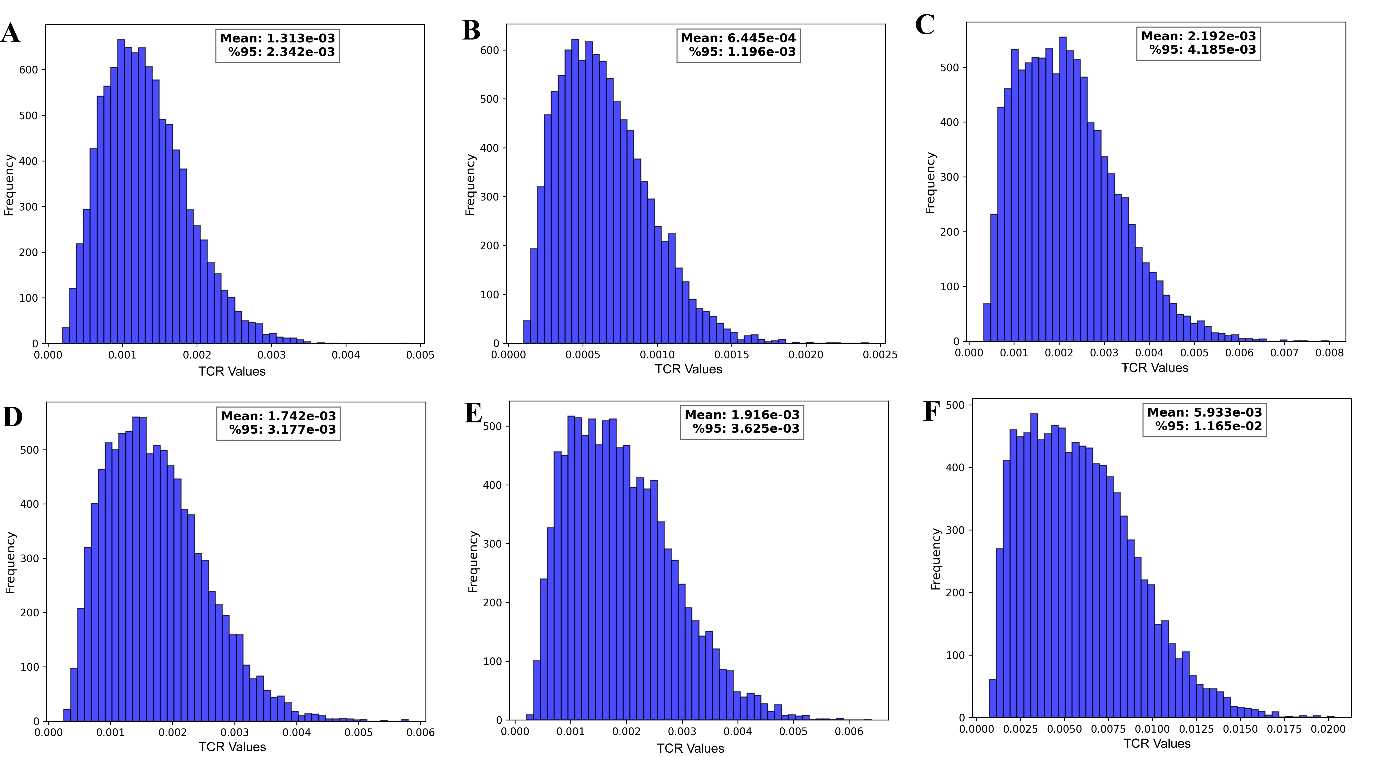
*

**Fig. S4.** Carcinogenic risk assessment in adults by monte carlo simulation. A) TCR-*Morchella importuna,* B) TCR-*Tricholoma scalpturatum,* C) TCR-*Infundibulicybe geotropa*, D) TCR-*Tricholoma populinum*, E) TCR-*Pholiota carbonaria,* F) TCR-*Laccaria laccata*

*
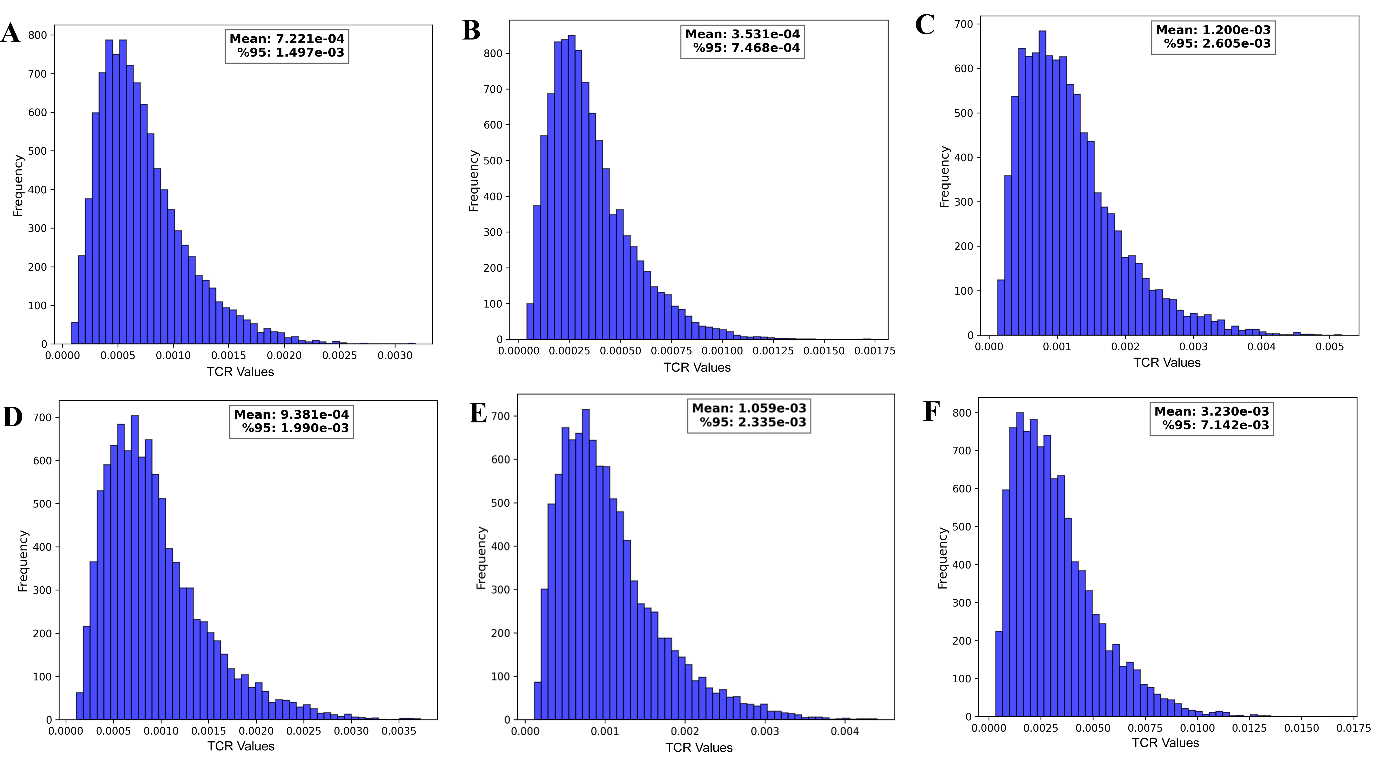
*

**Fig. S5**. Carcinogenic risk assessment in children by monte carlo simulation. A) TCR-*Morchella importuna,* B) TCR-*Tricholoma scalpturatum,* C) TCR-*Infundibulicybe geotropa*, D) TCR-*Tricholoma populinum*, E) TCR-*Pholiota carbonaria,* F) TCR-*Laccaria laccata*

*
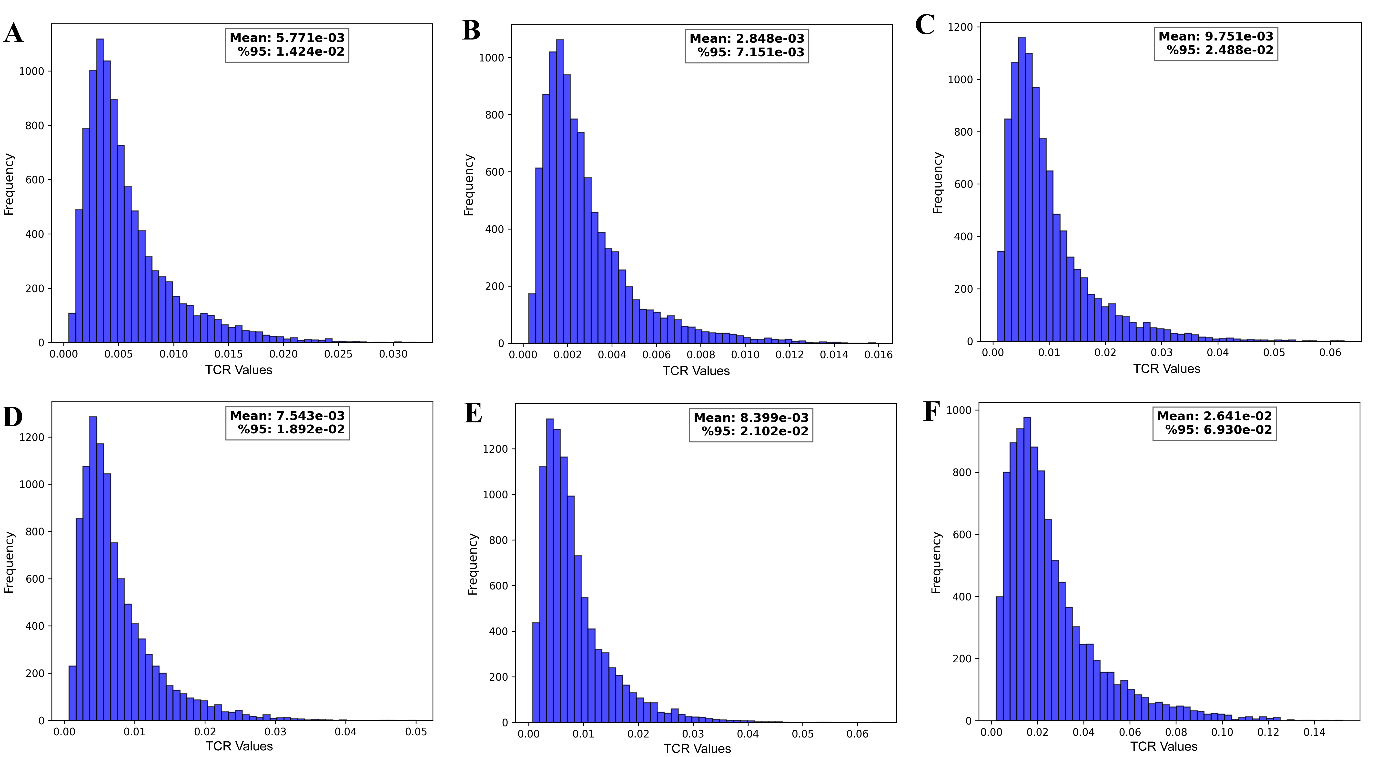
*

**Fig. S6.** Carcinogenic risk assessment throughout the life span by monte carlo simulation. A) TCR-*Morchella importuna,* B) TCR-*Tricholoma scalpturatum,* C) TCR-*Infundibulicybe geotropa*, D) TCR-*Tricholoma populinum*, E) TCR-*Pholiota carbonaria,* F) TCR-*Laccaria laccata*
